# Does menopause elevate the risk for developing depression and anxiety? Results from a systematic review

**DOI:** 10.1177/10398562231165439

**Published:** 2023-03-24

**Authors:** Salama Alblooshi, Mark Taylor, Neeraj Gill

**Affiliations:** School of Medicine and Dentistry, 97562 Griffith University, Gold Coast, QLD, Australia; School of Medicine and Dentistry, 97562 Griffith University. QLD, Australia; Mental Health Policy Unit, Health Research Institute, University of Canberra, ACT, Australia and School of Medicine and Dentistry, 97562 Griffith University, Gold Coast, QLD, Australia

**Keywords:** menopause, depression, anxiety, review, vasomotor symptoms

## Abstract

**Objective:**

To determine whether menopause elevates the risk for developing diagnostic depression and anxiety. Menopause-associated vasomotor symptoms such as insomnia and hot flushes are well recognized, but no systematic review of the psychological consequences of menopause has been undertaken. Menopause can be a time of social change for women, confounding any correlation.

**Methods:**

Using PRISMA methodology, we conducted a systematic review of all published (in English) original data examining a relationship between menopause and depression and anxiety. We ranked the quality of all included studies using Grading of Recommendations, Assessment, Development and Evaluation (GRADE) criteria.

**Results:**

Twenty-two selected studies were summarized and compared, being eight cross-sectional surveys; one retrospective cohort, and 13 prospective cohort studies. Depression and anxiety are common during menopause and the post-menopause, with vasomotor symptoms and a prior history of major depression elevating risk of menopausal associated depression. Psychosocial factors also may increase risk of depression during menopause.

**Conclusions:**

Menopause increases vulnerability to depression and anxiety, perhaps via estrogen fluctuations affecting serotonin and GABA. Underlying neuroticism and contemporaneous adverse life events are also risk factors for menopausal decompensation with depression.

Menopause, or *menstrual-pause*, refers to the inevitable point in a woman’s life when ovulation ceases and the production of estrogen and progesterone falls and stops. Most women reach menopause at an average age of 51–52 years.^1^

Menopause occurs in three stages: perimenopause, menopause, and the post-menopause. A woman is “post-menopausal” after 12 months of no menstrual cycle or period.^2^ Psychological symptoms include mood dyregulation, loss of libido, anxiety, irritability, sleeping difficulties, forgetfulness, and trouble concentrating or making decisions.

Perhaps surprisingly, no review of whether menopause elevates the risk of developing major depression and/or anxiety has previously been undertaken.

We conducted a systematic review to determine the influence of the menopausal transition on mood and anxiety and provide guidance on the associated risks for menopause-related anxiety and depression.

## Method

This systematic review is registered with PROSPERO (CRD 4202233498). The literature search was performed according to the PRISMA guidelines for systematic reviews. Two reviewers independently searched databases including PubMed, Cochrane library, Medline, Embase, PsychINFO, AMED, and Elsevier science from the earliest record to 10 December 2021.

The following terms were used: “depression,” “depressive disorder,” “anxiety,” “mood disorder,” “menopause,” and “menopausal transition.” In addition, cross-references in the included studies were hand-searched to identify additional eligible original studies.

Articles were selected based on a priori inclusion and exclusion criteria. A study was considered eligible if it included original data on women undergoing menopausal transition, with a clearly described measure of depression or an anxiety disorder.

**Figure 1.**
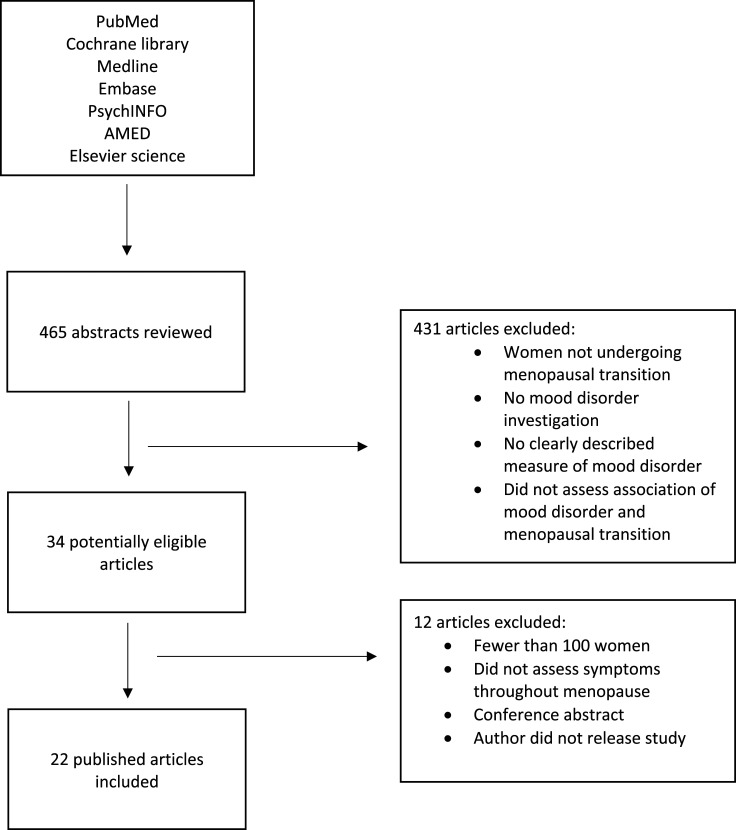
PRISMA flow chart.

Studies that did not evaluate women’s mental state during menopause or enrolled fewer than 100 participants and did not assess the association between menopause and mental health were excluded from the study. Non-English language studies and opinion pieces or reviews were also excluded (Figure 1).

All eligible studies were independently reviewed by two authors and the studies that satisfied the inclusion criteria were summarized into a standardized table. Every included study was independently rated for quality, using the Grading of Recommendations, Assessment, Development and Evaluation (GRADE) criteria.

**Table 1.** Summary of included studies

Study	Date published	Location of study	Study design	Number of participants	Psychiatric history assessment?	Quality rating (GRADE)	Key results
Almeida et al.	2016	Perth, Australia	Cross-sectional survey	1612	Yes	Low	Non-significant excess of depressive symptoms during transition and early post-menopause.
Amore et al.	2004	Italy	Cross-sectional	1434	Yes	Low	Depressive symptoms lower in pre-menopausal than post-menopause. Depression and significant anxiety present throughout and after menopause. Prior depression predicts subsequent post-menopausal depression.
Amore et al.	2007	Italy	Cross-sectional survey	1345	Yes	Low	Depressive and sexual symptoms more severe in the post-menopausal group, and strongly associated with life events.
Annivero et al.	2017	Milan, Italy	Prospective cohort	156	Yes	High	No significant difference across the menopause
Chou et al.	2015	Tainan City, Taiwan	Cross-sectional survey	111	Yes	Low	Depressive symptoms during the menopausal transition predicted depressive symptoms over 30 months. However, after controlling for it, the previous diagnosis of major depressive disorder menopausal status and vasomotor symptoms did not predict depressive symptoms over 30 months whereas neuroticism did.
Filipa et al.	2012	Portugal	Cross-sectional	992	Yes	Low	All symptoms in peri- and post-menopausal women elevated risk compared to pre-menopause. Peri-menopausal women did not differ from post-menopausal regarding perceived loss of control, anxiety or depression.
HUNT-II study	2009	Norway	Prospective cohort	16,080	No	Moderate	Hospital anxiety and depression scale-Anxiety peaked in perimenopause. High anxiety connected to hormone fluctuation.
Juang et al.	2005	Taiwan	Cross-sectional survey	1273	No	Low	Depression and anxiety were not associated with menopausal status but with hot flashes. After controlling for educational status and insomnia, anxiety and depression were significantly higher in peri- and post- menopausal women who experienced hot flashes.
Melbourne women’s midlife health project	2016	Melbourne, Australia	Cohort study	438	No	Moderate	Experience of negative mood and depressive symptoms was highest during the menopausal transition and lowest in the late post-menopause. Furthermore, increasing age was associated with reduction of depressive symptoms and negative mood. Yet when controlling for age, there was no difference found between early and late post-menopause stage.
Peking Union Medical College Hospital Aging Longitudinal Cohort of Women in Midlife (PALM) study	2019	Beijing, China	Prospective cohort	430	No	Low	Depression is more common than anxiety Both anxiety and depression are frequent in early years of menopause Poor health status and sleep independently associated with anxiety, whereas higher body mass index, poor physical health, low level of education, and night sweats independently associated with depression
Peking Union Medical College Hospital Aging Longitudinal Cohort of Women in Midlife (PALM) study	2020	Beijing, China	Prospective cohort	430	No	Low	Strong relationship between vasomotor and mood symptoms during menopause.
Personality and total health (PATH) through life project	2017	Canberra, Australia	Prospective cohort	711	Yes	Moderate	Higher risk of depression during perimenopause and symptoms of anxiety during post-menopause. In women with no history of depression or anxiety, the perimenopause and post-menopausal stages are associated with increased risk, relative to pre-menopause.
Penn ovarian aging study	2006	Pennsylvania, USA	Prospective cohort	231	Yes	Moderate	Menopause strongly associated with *de novo* depression in women with no prior history of depression.
Study of Women’s Health Across the Nation (SWAN)	2008	Pittsburgh, USA	Prospective cohort	226	Yes	Moderate	Earlier psychological problems and contemporary stressors more important than vasomotor symptoms for first episode of depression during menopause.
Study of Women’s Health Across the Nation (SWAN)	2012	Pittsburgh, USA	Prospective cohort	1970	No	Moderate	Women who undergo a hysterectomy with or without bilateral oophorectomy do not experience more depressive symptoms post-surgery.
Study of Women’s Health Across the Nation (SWAN)	2015	Pittsburgh, USA	Prospective cohort	443	Yes	Moderate	Women without history of depression at baseline at lower risk of developing depression than those with a prior history. For peri- and post-menopausal, prior anxiety was risk factors for depression.
Terauchi et al.	2012	Tokyo, Japan	Cross-sectional survey	237	No	Low	Insomnia is highly prevalent among peri- and post- menopausal women. Difficulty in initiating sleep and non-restorative sleep are significantly associated with anxiety and depression respectively.
The Harvard study of mood and cycles	2006	Massachusetts, USA	Prospective cohort	460	Yes	Moderate	Those with no history of depression who enter menopause early have a significant risk for new depression.
The Maccabi Healthcare Services (MHS) database study	2020	Israel	Retrospective cohort	17,051	Yes	Moderate	Menopausal symptoms are associated with increased burden of disease and healthcare utilization. Symptomatic peri- and post-menopausal women are more likely to have a higher prevalence of depression and anxiety in the year following their first menopause diagnosis.
The MIDUS study	2011	New Jersey, USA	Prospective longitudinal study	986	No	Moderate	Depressive symptoms predicted 9-year follow-up levels of menopausal symptoms controlling for depressive symptoms, age, financial status and education. Menopausal symptoms predicted 9-year follow-up levels of depressive symptoms controlling for menopausal symptoms, age, financial status and education.
The Zurich study	2016	Zurich, Switzerland	Prospective cohort	168	No	Low	Mental health problems between ages 41 and 50 not directly related to menopause
Ying et al.	2008	Beijing, China	Cross-sectional	1280	Yes	Low	Depression and anxiety common in menopause, related to psychosocial factors.

## Results

### Synthesis of results

#### Prevalence of depression and/or anxiety during the menopausal transition

Seven of the studies included here, namely, five cohort studies^3–7^ and two cross-sectional surveys,^8,9^ found a significant association between the menopausal transition and depression and/or anxiety. Five other studies didn’t (Table 1).^10–14^

The Study of Women’s Health Across the Nation^3^ found that being peri-menopausal confers a high risk for recurrence of major depressive disorder (MDD) but not for *de novo* MDD, relative to pre-menopause status. Being peri-menopausal compared to pre-menopausal more than doubled the risk of depression during follow-up.

The Penn Ovarian Aging Study^4^ noted a four-fold increase in depression in women with no history of depression during their menopausal transition compared to their pre-menopausal status. Moreover, a diagnosis of MDD was more than twice likely to occur in women with no history of pre-menopausal depression.

The PATH project revealed^5^ that being peri-menopausal was associated with a significantly increased risk of depressive symptoms relative to pre-menopause. Furthermore, being peri-menopausal was associated with an increased risk of depression and anxiety in women without history of probable depressive or anxiety disorder.

The Harvard study^6^ found that pre-menopausal women with no lifetime history of MDD were nearly twice as likely to develop peri-menopausal depressive symptoms compared to women with no history of depression. The Melbourne women’s midlife health project^7^ (MWMHP) also found that women in the menopausal transition and early post-menopausal phase were at higher risk of depressive symptoms and low mood than the late post-menopause.

Pimenta et al.^8^ showed that psychological symptoms were significantly elevated in peri-menopausal women compared to matched pre-menopausal women. Almeida et al.^9^ reported that reproductive status did not affect the prevalence of MDD, but when contrasted with pre-menopause, the peri-menopausal phase was associated with an elevated risk of developing depressive symptoms.

#### The prevalence of depression and/or anxiety after menopause

Five out of the twenty-two studies found a significant increased prevalence of depression and anxiety in post-menopausal women. A cross-sectional survey^15^ from Italy showed that depressive symptoms were significantly higher in the post-menopausal compared to the pre-menopausal women.

Almeida et al.^9^ reported that significantly more post-menopausal than pre-menopausal women had depressive symptoms, and the PATH project^5^ revealed that being post-menopausal was associated with increased risk of anxiety symptoms compared to pre-menopause.

Pimenta et al.^8^ noted adverse psychological symptoms were significantly elevated in post-menopausal women compared to their pre-menopausal counterparts.

In the Study of Women’s Health Across the Nation,^3^ being post-menopausal elevated the risk four-fold for major depressive *relapse* but did not significantly elevate risk for new MDD.

By contrast, Gibson et al.^16^ documented that in women who reached menopause, symptoms of anxiety and depression decreased in the years after the final menstrual period or hysterectomy.

#### Potential risk factors affecting mood, anxiety, and menopause

Biological risk factors have been shown in eleven studies here to play a role in the onset of depression and/or anxiety in the different menopausal stages. These biological factors include the vasomotor symptoms (VMS) of menopause, unrelated chronic medical diseases, and a history of premenstrual syndrome. Seven studies reported that depression and anxiety were strongly associated with VMS and related poor sleep.^3,10,13,17–20^

A prospective longitudinal study^19^ from New Jersey, USA, suggested that depressed women had more difficulty coping with the symptoms and physical changes of menopausal transition, and that those who experience more severe menopausal symptoms were more likely to have depressive symptoms during the menopausal transition.

Juang et al.^21^ noted a significant relationship between depression and anxiety, and the presence of hot flushes (part of VMS) in both peri- and post-menopausal women. This association remained significant even after educational status and insomnia were controlled for.

Chronic medical diseases and a decrease in physical health have also been associated with mood symptoms during menopause.^3,8–10^ Having at least one chronic medical condition prior to study entry more than doubled the risk of depression. Impaired role functioning because of physical health problems increased the risk of developing depression^3^ by 88%.

Psychological factors that played a role in the onset of new mood disorder in menopause include a past history of depression and anxiety,^3,9,12,15,17,22^ a history of postpartum depression,^9^ and an adverse perception of menopause.^22^ A past history of depression or anxiety was also a significant predictor of the onset of depressive symptoms during the menopausal stages. Women with a history of depression or anxiety were more prone to develop mood symptoms during menopause.^3,9,12,15,17,22^ A history of anxiety also increased the risk of development of depressive symptoms during menopause.^3,9^

The prospective long-term Zurich study^12^ demonstrated that neuroticism (a personality trait disposition to negative mood and anxiety) at age 30 significantly predicted increased prevalence of major depression and anxiety disorders during menopause. Chou et al.^17^ demonstrated that regardless of age, neuroticism was found to be significantly associated with depressive symptoms during the menopause transition.

Numerous studies have demonstrated the relationship between social factors and mood disorders during menopause, such as age, stressful life events, marital status, low socio-economic or financial status, and lack of family or social support.^3,6–9,12,13,23^ These stressful life events included decreased sexual activity; and children failing to enter college or get a job.^8,13^ Anxiety and depression were also more severe in post-menopausal women from rural or low socio-economic areas.^23^

## Discussion

### Main findings

This review revealed that during the menopausal transition, symptoms of anxiety and depression are common. We have ranked the main risk factors for depression and anxiety during menopause, as well as any protective factors in the table below.

**Table 2.** Risk and protective factors for developing menopausal depression or anxiety

Major risk factors	Minor risk factors	Protective factors
Vasomotor symptoms	Lack of social support	Social support/positive affirmations
History of major depressive disorder	Single or divorced	Menopausal hormone therapy
Neuroticism	Negative perception of aging or menopause	Counselling/psychological therapy
Stressful life events	History of premenstrual syndrome/premenstrual dysmorphic disorder	Healthy lifestyle including exercise
Low financial or educational status		Meditation/mindfulness

One of the biggest single risk factors for menopausal associated depression was a previous history of depressive illness, that is, a relapse of pre-existing depression. Vasomotor symptoms such as insomnia and hot flushes were highly correlated with both new incident depression and anxiety, and a relapse of prior depressive illness.

Both contemporaneous adverse social factors or life events and an underlying predisposition to depression and anxiety (neuroticism) were also found to elevate the risk of developing depression during menopause.

### Causation

It has been suggested that changes in hormonal levels during the menopausal transition might influence the brain via hypothalamic and hippocampal function. Steroid hormones affect serotonin and GABA signaling, which along with fluctuating neuronal opioids during menopause have been associated with depression, irritability, and anxiety.^24,25^ However, one study we included noted a decline in anxiety and depressive symptoms during the post-menopausal stage after bilateral oophorectomy (surgical menopause).

Menopausal vasomotor symptoms including hot flushes and sleep disturbance were found in this review to be associated with depression and anxiety during menopause, although the underlying mechanism remains unclear. Hot flushes are thought to occur as a result of the dysregulation in the thermoregulatory center in the hypothalamus, which happens during ovarian failure and estrogen withdrawal. These hot flushes are associated with sleep disturbance and can adversely affect mood.^26^ Reduced estrogen levels and sleep disturbance are also similarly seen during post-natal depression.

Compared to women with no previous depressive disorder, increased variability of follicle-stimulating hormone (FSH) and estrogen, and decreased levels of inhibin B and increased levels of luteinizing hormone are linked to depression in women with a previous history of depressive disorder.^27^

Neuroticism, a trait disposition to experiencing negativity,^28^ predicts depression during menopause. People with elevated neuroticism can find minor frustrations overwhelming. Furthermore, a negative perception towards menopause seems to play a significant role in predicting depression and anxiety during menopause. Stressful life events during menopause, such as illness, marital discord, and children leaving home or failing to enter college or getting a job, are all associated with depression and anxiety during menopause.

### Strengths and limitations of this review

Some of the included studies did not assess the presence of a past psychiatric history in women during menopause, which we found to be a major risk factor. Also, numerous studies had little or no data on anxiety during menopausal transition. Finally, almost all the cross-sectional studies assessed mood through self-report surveys or questionnaires, leading to potential bias or misattribution, and highlights concerns about the consistent diagnostic validity for major depressive disorder.

The selected articles had a wide range of study methods, including cohort studies of several years’ duration originating from many countries, and included studies examined the influence of diverse biopsychosocial factors. Stringent inclusion and exclusion criteria and a quality ranking allowed focus on the highest quality data.

### Conclusions and future research

Both the direct steroid hormone level fluctuations during menopausal transition and biopsychosocial factors during menopause are important in menopause-associated depression.

There is less data on menopausal-incident anxiety, but we cautiously suggest an evidence-based link between VMS, sleep disturbances, and anxiety associated with menopause.

Early screening and clinical intervention using the risks identified here could reduce the possibility of developing depressive symptoms during menopause.

In conclusion, there is a “window of vulnerability” for depression and anxiety during the menopause.
